# Supplementary material for: Unexpected genetic and microbial diversity for arsenic cycling in deep sea cold seep sediments
Source: NPJ Biofilms Microbiomes. 2023 Mar 29;9:13. doi: 10.1038/s41522-023-00382-8 (PMC10060404; doi:10.1038/s41522-023-00382-8)
Supplement: Supplementary file 1 — Supplementary Information [file 41522_2023_382_MOESM1_ESM.pdf]

Unexpected genetic and microbial diversity for arsenic cycling in deep sea cold seep sediments

Chuwen Zhang[1], Xinyue Liu[1, 2], Ling-Dong Shi[3], Jiwei Li[4],

Xi Xiao[5], Zongze Shao[1, 6], Xiyang Dong[1, 6] *,

[1] Key Laboratory of Marine Genetic Resources, Third Institute of Oceanography, Ministry of Natural Resources, Xiamen, China

[2] School of Marine Sciences, Sun Yat-Sen University, Zhuhai, China

[3] College of Environmental and Resource Sciences, Zhejiang University, Hangzhou, China

[4] Institute of Deep-Sea Science and Engineering, Chinese Academy of Sciences, Sanya, China

[5] Key Laboratory of Marine Mineral Resources, Ministry of Natural Resources, Guangzhou Marine Geological Survey, China Geological Survey, Guangzhou, China

[6] Southern Marine Science and Engineering Guangdong Laboratory (Zhuhai), Zhuhai, China

* Correspondence can be addressed to Xiyang Dong (dongxiyang@tio.org.cn).

Supporting Information

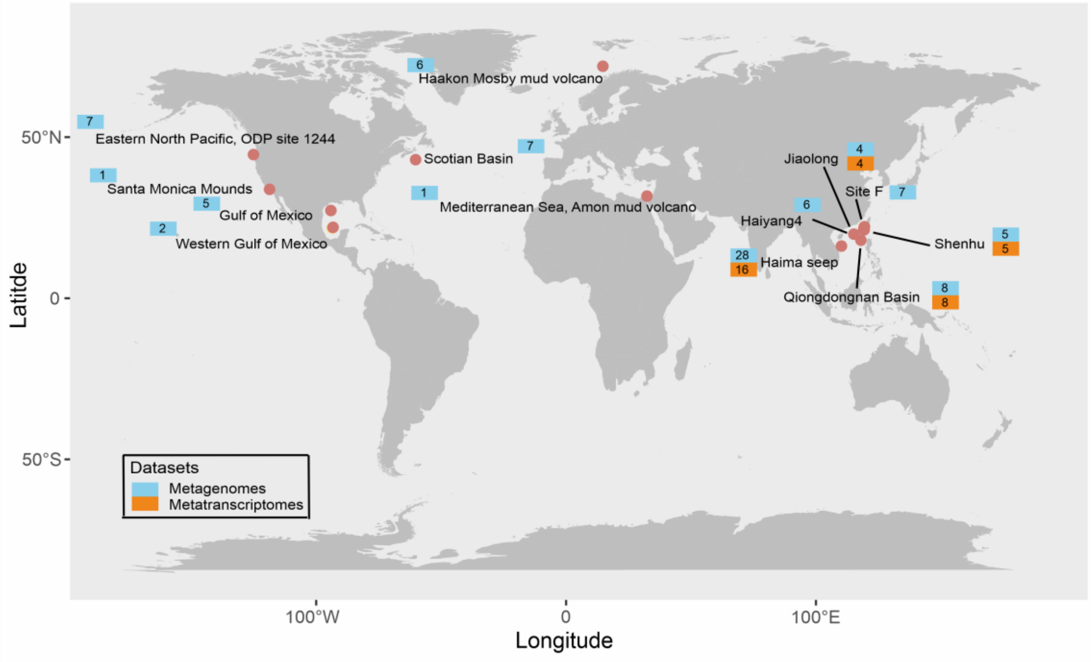

Supplementary Figure 1. Geographical distributions of 13 cold seep sites analyzed in this study. Colors indicate the type of samples collected at each site: metagenome (blue); metatranscriptome (orange). The numbers indicate the number of samples for metagenomes or metatranscriptomes. Further details for each metagenome and metatranscriptome can be found in **Supplementary Data 1**.

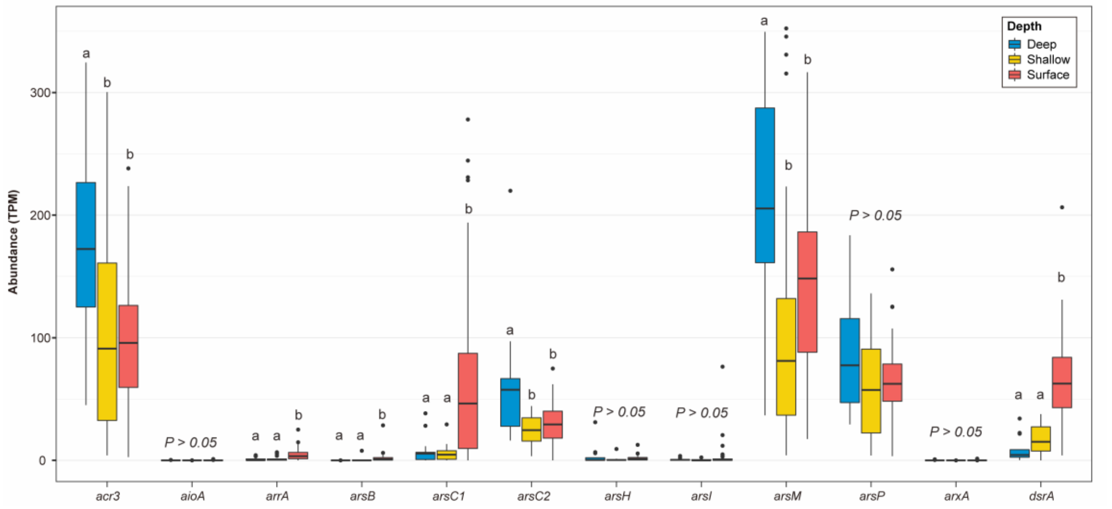

Supplementary Figure 2. The abundances of As-cycling gene across different sediment depths with *drsA* gene as a comparison. One-way analysis of variance (ANOVA) was performed for the comparison, followed by least significant difference (LSD) test. Boxplot components: center line, median values; box limits, upper and lower quartiles; whiskers, 1.5× interquartile range; points, outliers.

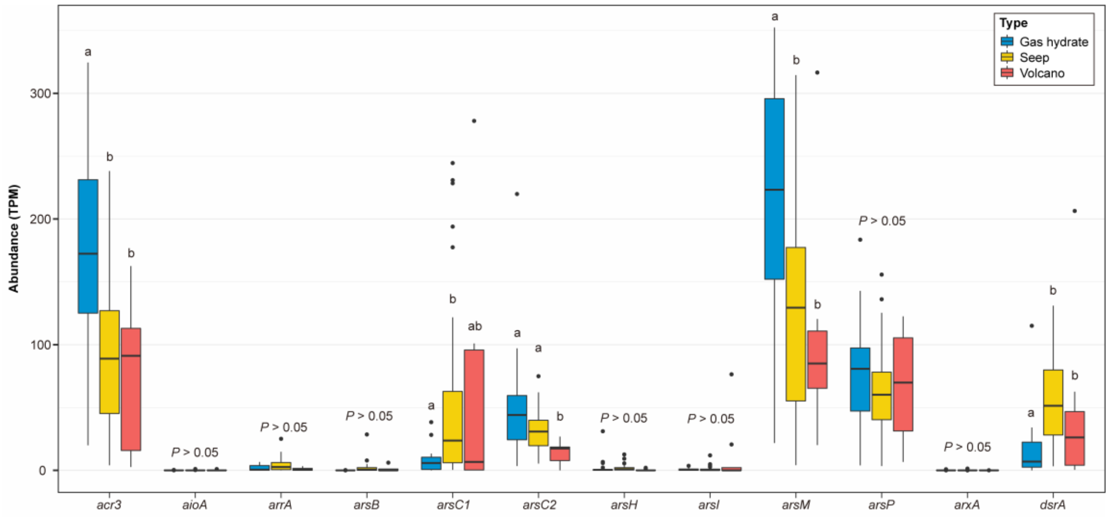

Supplementary Figure 3. The abundances of As-cycling gene across different types of cold seep ecosystems with *drsA* gene as a comparison. One-way analysis of variance (ANOVA) was performed for the comparison, followed by least significant difference (LSD) test. Boxplot components: center line, median values; box limits, upper and lower quartiles; whiskers, 1.5× interquartile range; points, outliers.

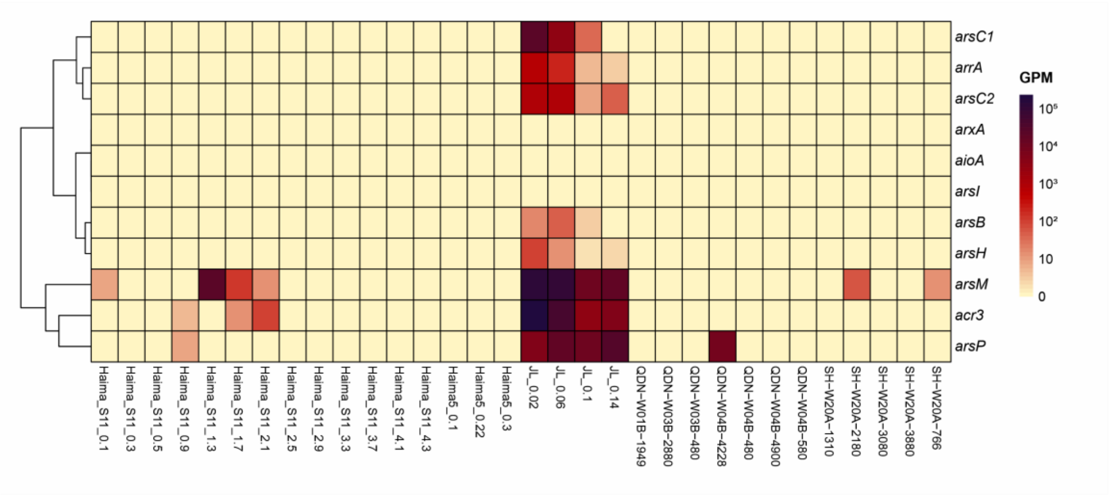

Supplementary Figure 4. The abundance of As-cycling transcripts in 33 sediment metatranscriptomes. The transcript abundance of each gene was normalized by the gene length and sequencing depth and represented as TPM (transcripts per million) value.

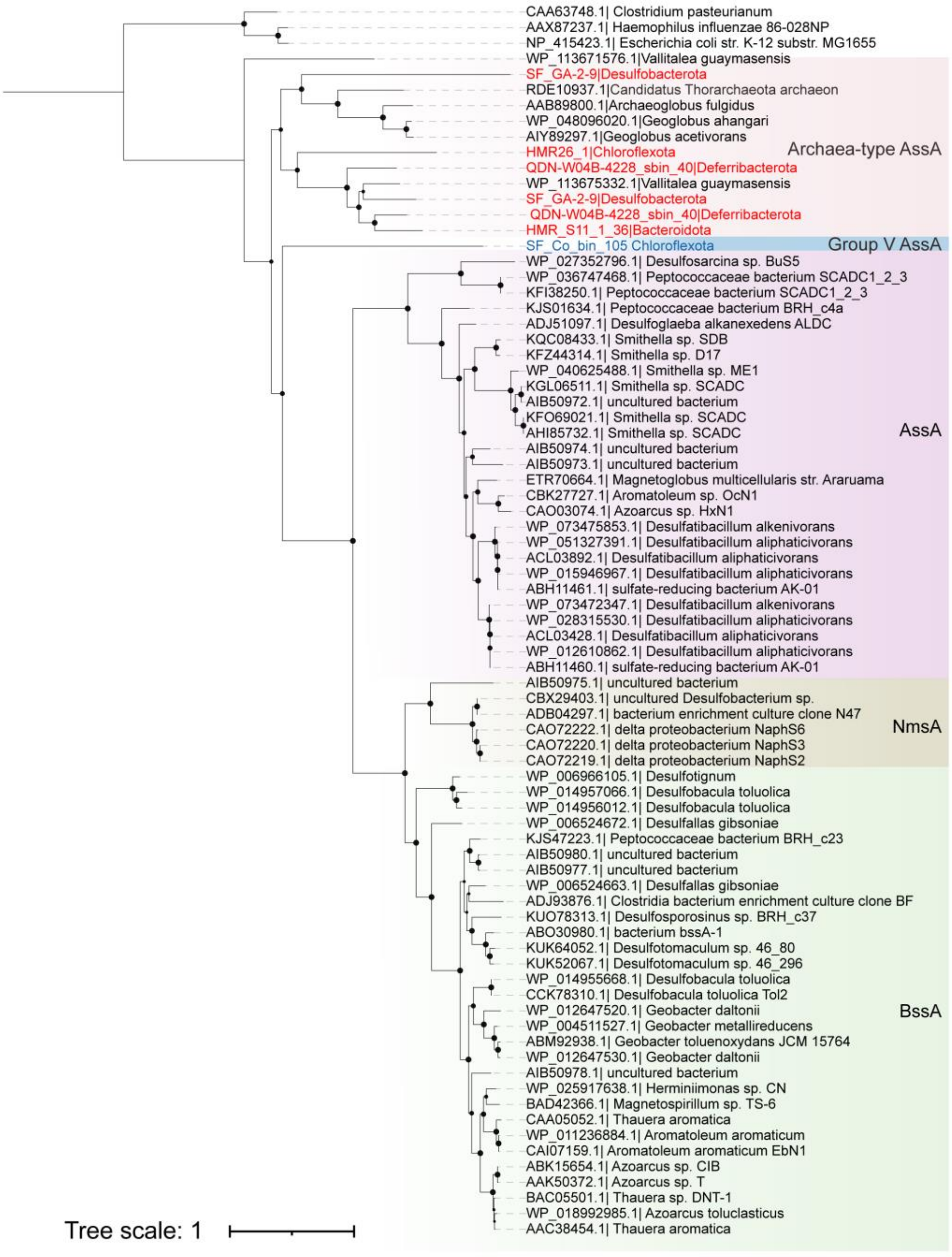

Supplementary Figure 5. Phylogenetic analysis of identified AssA protein sequences in *arrA*-carrying MAGs. Bootstrap values >70% are shown as black dots on branch nodes. Scale bar represents amino acid substitutions per site.
